# Macrolide resistance in *Mannheimia haemolytica* isolates associated with bovine respiratory disease from the German national resistance monitoring program GE*RM*-Vet 2009 to 2020

**DOI:** 10.3389/fmicb.2024.1356208

**Published:** 2024-03-01

**Authors:** Valeria Kostova, Dennis Hanke, Heike Kaspar, Stefan Fiedler, Stefan Schwarz, Henrike Krüger-Haker

**Affiliations:** ^1^Institute of Microbiology and Epizootics, Centre for Infection Medicine, School of Veterinary Medicine, Freie Universität Berlin, Berlin, Germany; ^2^Veterinary Centre for Resistance Research (TZR), School of Veterinary Medicine, Freie Universität Berlin, Berlin, Germany; ^3^Federal Office of Consumer Protection and Food Safety (BVL), Berlin, Germany

**Keywords:** *Pasteurellaceae*, antimicrobial resistance (AMR), integrative and conjugative element (ICE), whole-genome sequencing (WGS), cattle

## Abstract

Data collected from the German national resistance monitoring program GE*RM*-Vet showed slowly increasing prevalence of macrolide resistance among bovine respiratory disease (BRD)-associated *Pasteurellacae* from cattle over the last decade. The focus of this study was to analyze the genetic basis of antimicrobial resistance (AMR) and the prevalence of multidrug-resistance (MDR)-mediating integrative and conjugative elements (ICEs) in 13 German BRD-associated *Mannheimia haemolytica* isolates collected between 2009 and 2020 via whole-genome sequencing. Antimicrobial susceptibility testing (AST) was performed via broth microdilution according to the recommendations of the Clinical and Laboratory Standards Institute for the macrolides erythromycin, tilmicosin, tulathromycin, gamithromycin, tildipirosin, and tylosin as well as 25 other antimicrobial agents. All isolates either had elevated MICs or were resistant to at least one of the macrolides tested. Analysis of whole-genome sequences obtained by hybrid assembly of Illumina MiSeq and Oxford Nanopore MinION reads revealed the presence of seven novel Tn*7406*-like ICEs, designated Tn*7694*, and Tn*7724*- Tn*7729*. These ICEs harbored the antimicrobial resistance genes *erm*(T), *mef* (C), *mph*(G), *floR, catA3, aad(3“)(9), aph(3‘)-Ia, aac(3)-IIa, strA, strB, tet*(Y), and *sul2* in different combinations. In addition, mutational changes conferring resistance to macrolides, nalidixic acid or streptomycin, respectively, were detected among the *M*. *haemolytica* isolates. In addition, four isolates carried a 4,613-bp plasmid with the β-lactamase gene *bla*_ROB − 1_. The detection of the macrolide resistance genes *erm*(T), *mef* (C), and *mph*(G) together with other resistance genes on MDR-mediating ICEs in bovine *M. haemolytica* may explain the occurrence of therapeutic failure when treating BRD with regularly used antimicrobial agents, such as phenicols, penicillins, tetracyclines, or macrolides. Finally, pathogen identification and subsequent AST is essential to ensure the efficacy of the antimicrobial agents applied to control BRD in cattle.

## Introduction

*Mannheimia haemolytica* is a Gram-negative, facultatively pathogenic bacterium of considerable importance in the development of the multifactorial bovine respiratory disease (BRD). The bacterium often remains in the tonsillar crypts of healthy bovines, however, stress or viral infections can lead to rapid growth, followed by manifestation in the lungs that often results in acute fibrinous pleuropneumonia (Singh et al., 2011). The importance of BRD lies not only in the high morbidity, but also in the enormous economic losses, as it is one of the costliest diseases among feedlot cattle (Cortes et al., 2021). Germany is the largest milk producer in the European Union and the second largest producer of beef and veal following France. In May 2023, Germany had a cattle population of around 11 million animals. On average, a German cattle farm keeps 104.4 animals. More than three quarters of cattle live on farms with at least 100 animals. However, only 2% of the German cattle farms that keep male animals older than one year for beef production have herd sizes of 100 or more animals. Instead, most of these farms (75.2%) keep only a small number of up to nine animals. In Germany, nine out of 10 cattle are kept in loose-housing barns in which they can move around freely. The design of these stables varies considerably: Beef cattle are mainly kept in enclosed or partially enclosed barns with concrete slatted floors, while dairy cows are usually kept in spacious cubicle barns. Moreover, cattle may be found in seasonal or year-round tethered housing in smaller farms. Regardless of the type of housing in the barn, around one in three cattle have regular access to pasture in summer.^1^ In contrast, the world's largest beef exporters, Brazil, Australia,^2^ and the USA, generally use feedlots—large, fenced areas of barren land—as their main housing system for fattening cattle, with significantly larger herd sizes of usually several thousands of animals (Andrade et al., 2020; Koyun et al., 2023). Controlling diseases, such as BRD, can be especially difficult in these herds with huge numbers of animals living together (Koyun et al., 2023).

Due to the use of antimicrobial agents, such as phenicols, penicillins, tetracyclines, and macrolides, for treating BRD, the risk of *M. haemolytica* developing and/or acquiring antimicrobial resistance (AMR) mechanisms is high (Watts and Sweeney, 2010; de Jong et al., 2014). Macrolides are ribosome-targeting antimicrobial agents with a 13-, 14-, 15- or 16-membered lactone ring. They interfere with bacterial protein synthesis by binding to the 50S ribosomal subunit. Substances like tulathromycin (a 9:1 mixture of 13- and 15-membered macrolides) or tilmicosin (a 16-membered macrolide) as well as tildipirosin (a 16-membered macrolide) and gamithromycin (a 15-membered macrolide) are approved for the treatment of BRD in Germany (Bäumer, 2012). So far, a variety of macrolide resistance mechanisms has been described in *M. haemolytica*, including (i) chemical target site modification of the ribosomal RNA (rRNA) conferred via methylases encoded by the genes *erm*(42) or *erm*(T), (ii) enzymatic inactivation of macrolides by phosphotransferases encoded by the genes *mph*(E) or *mph*(G), (iii) efflux of macrolides encoded by the genes *mef* (C) or *msr*(E) as well as (iv) mutational changes in one of two locations, A2058G or A2059G, of the 23S rRNA and, thereby, causing structural changes of the drug binding pocket and ultimately impairing drug efficacy (Vester and Douthwaite, 2001; Kadlec et al., 2011; Schink et al., 2022; Kostova et al., 2023). To date, macrolide-resistant *M. haemolytica* isolates have been frequently observed in North America (Klima et al., 2020). A recent study also showed the first macrolide-resistant bovine *M*. *haemolytica* isolate from Australia (Alhamami et al., 2021).

Giving consideration to the fact that multidrug resistance (MDR), which is defined as non-susceptibility to at least one agent in three or more antimicrobial classes (Sweeney et al., 2018), is described among BRD pathogens at increasing percentages, MDR is commonly mediated by integrative and conjugative elements (ICEs; Credille, 2020). ICEs are self-transferable mobile genetic elements (MGEs), which integrate site-specifically into the chromosomal DNA. They harbor core genes with essential functions for integration, excision, transfer and regulation, as well as accessory genes that may be beneficial for niche adaptation, such as AMR genes. During transmission, a circular intermediate is formed after excision from the chromosomal DNA, which then transfers conjugatively into another host cell and there eventually integrates into the chromosomal DNA (Johnson and Grossman, 2015). ICEs harboring the macrolide resistance genes *erm*(42) and/or *msr*(E)-*mph*(E) among other resistance genes have been frequently observed in BRD-associated *M. haemolytica* from North America during the last decade (Lubbers and Hanzlicek, 2013; Klima et al., 2014b; Clawson et al., 2016; Andrés-Lasheras et al., 2022). In Germany, two related MDR-mediating ICEs, designated Tn*7406* and Tn*7693*, have been reported in bovine *M. haemolytica* in the last two years. Tn*7406* carried only the macrolide resistance genes *mef* (C)-*mph*(G) in combination, while Tn*7693* additionally harbored the macrolide-lincosamide-streptogramin B-resistance gene *erm*(T) (Schink et al., 2022; Kostova et al., 2023).

Recent data from the German national resistance monitoring program GE*RM*-Vet have shown slowly increasing numbers of macrolide-resistant bovine *M. haemolytica* isolates since 2009. In this study, 13 macrolide-resistant *M. haemolytica* isolates from diseased cattle included in GE*RM*-Vet were further investigated to gain insight into their phylogenetic relationships and to elucidate the genetic basis of their AMR properties.

## Materials and methods

### Bacterial isolates

A total of 13 *M. haemolytica* isolates were included in the study. All isolates were obtained from cattle suffering from BRD [calves < 6 months (*n* = 7), adult cattle (*n* = 6)]. The isolates originated from local diagnostic facilities in Germany and were included in the national resistance monitoring program GE*RM*-Vet during the years 2009 to 2020. The selection for our study was based on the isolates' phenotypic AMR profiles. Isolate 9, carrying the ICE Tn*7693*, has been the subject of a previous study (Kostova et al., 2023) that described the first detection of the *erm*(T) gene in *M*. *haemolytica*.

### Antimicrobial susceptibility testing (AST)

AST was carried out according to the recommendations of the Clinical and Laboratory Standards Institute (CLSI, 2023; Feßler et al., 2023). All 13 *M*. *haemolytica* isolates were subjected to broth microdilution using commercially available microtiter plates (MICRONAUT-S Large Animal, MERLIN GmbH Bornheim-Hersel, Germany; MICRONAUT-S, Small Animal, MERLIN GmbH Bornheim-Hersel, Germany) as well as microtiter plates which have been designed specifically for the German national resistance monitoring program GE*RM*-Vet^3^ (Sensititre, Thermo Fisher Scientific, Waltham, MA, USA). In total, MIC values were obtained for 31 antimicrobial agents, including β-lactams (amoxicillin/clavulanic acid 2:1, ampicillin, penicillin, cefoperazone, cefotaxime, ceftiofur, cefquinome, cephalexin and imipenem), tetracyclines (tetracycline, doxycycline), a polymyxin (colistin), a lincosamide (clindamycin), a pleuromutilin (tiamulin), phenicols (florfenicol, chloramphenicol), macrolides (erythromycin, gamithromycin, tildipirosin, tylosin, tilmicosin, and tulathromycin), aminoglycosides (gentamicin, neomycin, streptomycin), fluoroquinolones (enrofloxacin, marbofloxacin, ciprofloxacin, and nalidixic acid), and folate pathway inhibitors (sulfisoxazole, trimethoprim/sulfamethoxazole 1:19). When clinical breakpoints were provided in the aforementioned CLSI document, isolates were classified as susceptible, intermediate, or resistant. The functionality of the sulfonamide resistance gene *sul2* present in some of the isolates could not be investigated using the microtiter plate layouts described above. Therefore, we performed additional broth macrodilution tests for the sulfonamide sulfisoxazole according to CLSI recommendations (CLSI, 2023). In order to screen for inducible macrolide/lincosamide resistance, a D-zone test was performed according to CLSI recommendations (CLSI, 2023) using disks containing erythromycin (15 μg; BBL™ Sensi-Disc™, BD, Franklin Lakes, NJ, USA) and clindamycin (2 μg; Oxoid, Thermo Fisher Scientific, Waltham, MA, USA).

### Whole-genome sequencing (WGS)

#### DNA extraction

Genomic DNA of the 13 *M*. *haemolytica* isolates was extracted using the phenol-chloroform extraction method with some adjustments for *M*. *haemolytica*. A suspension of *M*. *haemolytica* in brain-heart-infusion (BHI) broth was incubated for 4 h at 37°C and 150 rpm in a shaking incubator. Then, 1.5 mL of the culture was transferred into a safe seal Eppendorf tube and centrifuged at 7.8 × *g* for 10 min. The supernatant was discarded and the procedure was repeated if the pellet was either too small or not well-defined. The pellet was resuspended in 1 mL of TES buffer and centrifuged at 7.8 × *g* for 10 min. The supernatant was discarded again and the pellet was resuspended in 500 μL of TES buffer. Then, 10 μL of 20% sodium dodecyl sulfate (SDS) were added to the tube, and the tube was gently inverted 10–12 times until the mixture became viscous. The mixture was incubated for 10 min at room temperature. Next, 600 μL ROTI^®^ Phenol/Chloroform/Isoamyl alcohol (25:24:1; Carl Roth GmbH + Co. KG, Karlsruhe, Germany) were added and the mixture was vigorously shaken for 5 min. Thereafter, the mixture was centrifuged at 16.2 × *g* for 10 min. After this, the upper aqueous phase was carefully transferred into a new sterile 1.5 mL Eppendorf tube. Then, 600 μL of 99.9% isopropanol were gently added and the tube was inverted slowly for 2 min. The DNA was pelleted by centrifugation at 16.2 × *g* for 2 min. The supernatant was decanted and 1 mL of 70% ethanol was poured over the pellet. Again, the tube was centrifuged at 16.2 × *g* for 1 min. The supernatant was removed and the DNA pellet was left to dry thoroughly upside down for at least 20 min at room temperature. Thereafter, the pellet was resuspended in 50 μL sterile demineralized water and 1 μL of 20 mg/mL RNase was added to the suspension. Finally, the DNA was left to soak overnight in a light-protected box at room temperature. DNA quality was checked with the NanoDrop^TM^ 1000 spectrophotometer (Thermo Fisher Scientific, Waltham, MA, USA) and the Qubit^TM^ 2.0 fluorometer (Thermo Fisher Scientific, Waltham, MA, USA). In addition, the DNA was visualized via agarose gel electrophoresis (1% TBE, 80 V, 90 min).

#### Short-read and long-read sequencing

All samples were subjected to short-read and long-read sequencing. From the extracted DNA, 1 ng was used for short-read sequencing, libraries were prepared using the Nextera XT DNA Library Preparation Kit (Illumina, Inc., San Diego, USA) according to the manufacturer's recommendations. The 2 × 300-bp paired-end sequencing in 40-fold multiplexes was performed on the Illumina MiSeq platform with the MiSeq reagent kit v3 (Illumina, Inc., San Diego, USA). Long-read sequencing was performed for 10 h with the Oxford Nanopore MinION device (Oxford Nanopore Technologies, Oxford, UK) using 400 ng of the same extracted DNA to generate a barcoded MinION one-dimensional library with the SQK-RBK004 kit. Barcoded DNA was pooled and loaded onto a R9.4.1 flow cell to carry out multiplexed sequencing.

#### Sequence assembly and annotation

For Illumina short-reads, Trim Galore v0.6.10 (RRID: SCR_011847) and FastQC v0.12.1^4^ were used for adapter trimming and quality check. Reads for all Nanopore data sets were basecalled and demultiplexed with Guppy basecaller v6.5.7 (Oxford Nanopore Technologies, Oxford, UK) into quality-tagged sequence reads (4,000 reads per fastq-file). Porechop v0.2.4^5^ and Filtlong v0.2.1^6^ were used for adapter trimming, elimination of reads below 1,000 bp or sequence quality worse than QV 7.5. LongQC^7^ was used for the quality check. Unicycler v0.4.9 (Wick et al., 2017) and the Flye algorithm in MaSuRCA v4.1.0 (Zimin et al., 2017) were used for hybrid assembly with MinION long-reads and Illumina short-reads to generate a closed genome. In addition, a third assembly was performed with Flye v2.9.2 (Kolmogorov et al., 2020) using the MinION long-reads, which were polished with NextPolish v1.4.1 (Hu et al., 2019) using Illumina short-reads. The three resulting closed, complete genomes were used to generate a corrected consensus sequence with Geneious v11.1.5 (Biomatters, Ltd., Auckland, New Zealand), which was annotated with RAST (Aziz et al., 2008; Overbeek et al., 2014; Brettin et al., 2015), Prokka v1.14.5 (Seemann, 2014), and Bakta (Schwengers et al., 2021). Isolate IDs, background information on the isolates' origin and sequencing metrics including the individual NCBI GenBank accession numbers of the obtained whole-genome sequences are given in Supplementary material 1. Transposon designations were provided by the transposon registry^8^ (Tansirichaiya et al., 2019).

### Serotyping, multi-locus sequence typing (MLST), and phylogenetic analysis

For all whole-genome sequences, serotyping and MLST were carried out via PubMLST^9^ (Jolley et al., 2018; Christensen et al., 2021). For the phylogenetic analysis of all *M*. *haemolytica* isolates, we generated a separate core-genome for MLST sequence types ST1 and ST4, respectively, with references from NCBI^10^ (eleven ST1 references, 13 ST4 references) and the bacterial pangenome analysis pipeline Panaroo (Tonkin-Hill et al., 2020) with default settings. With SNP-sites (Page et al., 2016), single nucleotide polymorphisms (SNPs) were extracted from the filtered core-genome alignments from Panaroo. With the extracted SNPs, IQ-Tree was used to generate phylogenetic maximum likelihood trees with the best model and 10,000 bootstraps (Minh et al., 2013; Nguyen et al., 2015; Kalyaanamoorthy et al., 2017), which were visualized with FigTree v1.4.4.^11^

### Investigation of AMR properties and ICEs

For identification of AMR genes, we used ABRicate^12^ with the NCBI AMRFinderPlus (Feldgarden et al., 2019) and ResFinder (Bortolaia et al., 2020) databases. By using the ISfinder tool^13^ (Siguier et al., 2006), mobile elements were detected. All results were verified with Geneious v11.1.4 (Biomatters Ltd., Auckland, New Zealand). Further sequence analysis and detection of Tn*7406*-like ICEs as well as AMR-mediating mutational changes was carried out using MAFFT alignment in Geneious v11.1.4 (Biomatters Ltd., Auckland, New Zealand).

## Results

### Phenotypic AMR profiles of *M. haemolytica*

**Figure 1:**
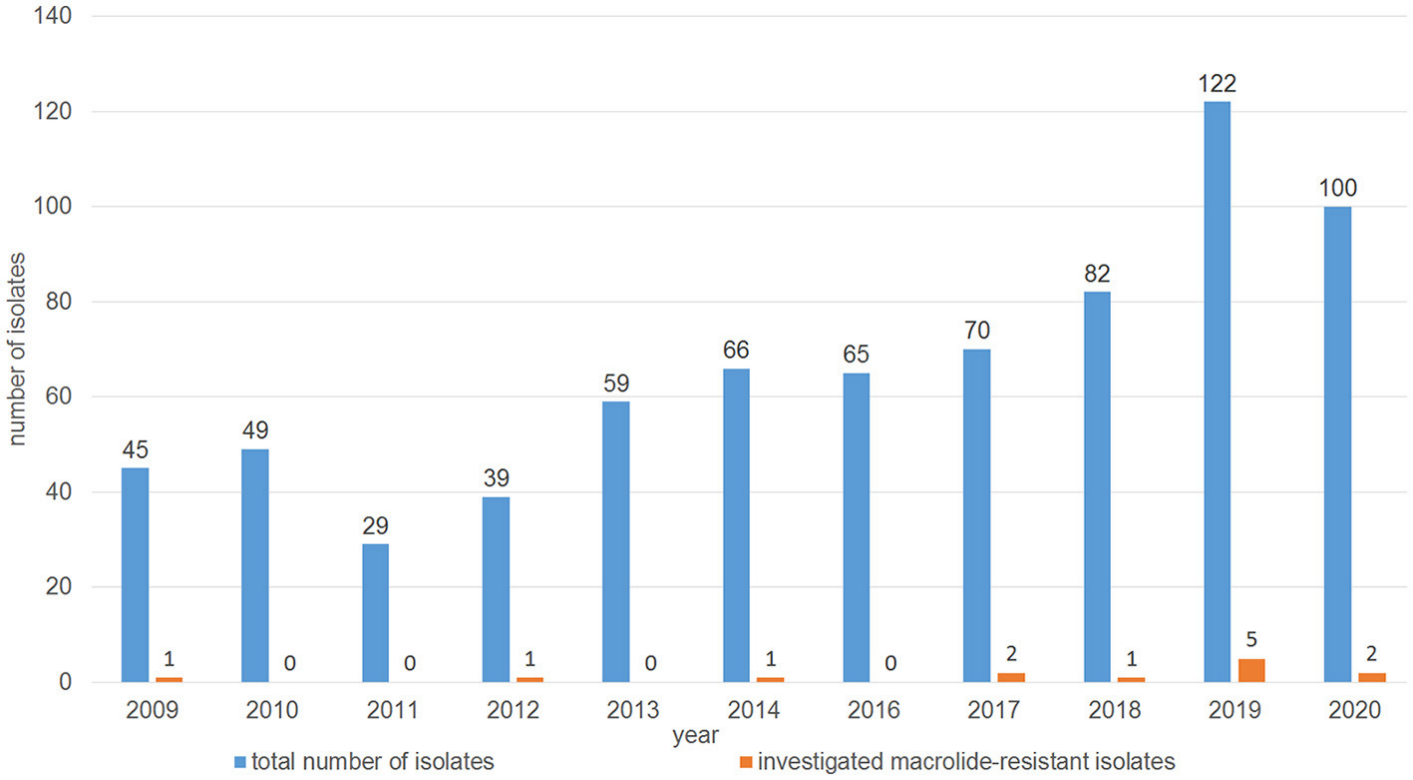
Distribution of the investigated macrolide-resistant *M*. *haemolytica* isolates among the *M*. *haemolytica* isolates collected in GE*RM*-Vet from cattle with respiratory disease between 2009 and 2020.

The distribution of minimal inhibitory concentration (MIC) data is shown in Table 1. All 13 isolates either showed elevated MICs or were resistant to at least one or more of the macrolides tested. More precisely, 10 isolates were resistant to tilmicosin (≥32 mg/L), with another two isolates being tilmicosin-intermediate (MIC 16 mg/L) and one isolate being susceptible (MIC ≤ 8 mg/L). Eleven isolates were classified as tulathromycin-resistant with MICs of ≥ 64 mg/L. These latter 11 isolates were also gamithromycin-resistant (MIC ≥ 16 mg/L), and exhibited elevated MICs of erythromycin (MIC ≥ 64 mg/L). In total, eight isolates showed resistance to tildipirosin with MICs of ≥ 64 mg/L. All 13 isolates exhibited elevated tylosin MICs of ≥ 64 mg/L. Furthermore, eight isolates exhibited elevated clindamycin MICs of ≥ 128 mg/L and six isolates had elevated tiamulin MICs of ≥ 32 mg/L. Four isolates exhibited resistance to the β-lactams penicillin and ampicillin. With regard to resistance to phenicols, seven isolates were resistant to florfenicol with MICs of ≥ 8 mg/L excluding another two isolates being intermediate (MIC 4 mg/L). Moreover, all but two isolates showed elevated MICs of chloramphenicol (MIC ≥ 16 mg/L). Resistance to tetracycline was detected in 11 isolates (MIC ≥ 16 mg/L). The resistance profiles for the aminoglycosides varied, with all isolates exhibiting elevated MIC values of 32 - ≥ 512 mg/L for streptomycin, while only six isolates had elevated MICs of gentamicin (4 - ≥ 16 mg/L). Elevated MICs of neomycin, observed in eight isolates, ranged between 16 and ≥ 64 mg/L. In terms of resistance to (fluoro)quinolones, all isolates were susceptible to enrofloxacin, marbofloxacin and ciprofloxacin, but seven isolates showed elevated nalidixic acid MICs. All 13 isolates exhibited elevated sulfisoxazole MICs of ≥ 256 mg/L, however, 11 of these isolates were susceptible to the combination of trimethoprim-sulfamethoxazole (MIC ≤ 0.125/2.375 mg/L), while two isolates showed MICs of 0.5/9.5 and ≥ 4/74 mg/L, respectively. Overall, 10 of the 13 isolates showed a phenotypic MDR profile. Among those antimicrobial agents, for which clinical breakpoints were available, the most prevalent AMR phenotype was 84.62% (11 of 13 isolates) for tetracycline, 61.54% (eight of 13 isolates) for gamithromycin, tildipirosin, tilmicosin, and tulathromycin, followed by 53.85% (seven of 13 isolates) for florfenicol and 30.77% (four of 13 isolates) for penicillin and ampicillin. In addition, we observed that macrolide resistance in the *M. haemolytica* isolates included in the GE*RM*-Vet program may have slowly increased during recent years (Figure 1). However, taking into account the total number of bovine *M*. *haemolytica* included in GE*RM*-Vet each year, macrolide resistance rates are still very low.

**Table 1:** Distribution of MIC values of the 13 *M*. *haemolytica* isolates included in this study.

**Antimicrobial agent(s)^b^**	**No. of isolates with MIC (mg/L)** ^**a**^																	
	**0.008**	**0.015**	**0.03**	**0.06**	**0.12**	**0.25**	**0.5**	**1**	**2**	**4**	**8**	**16**	**32**	**64**	**128**	**256**	**512**	**1,024**
Amoxicillin/ clavulanic acid (2:1)							13	0	0	0	0							
Ampicillin			7	2	0	0	0	0	0	0	0	0	4					
Penicillin				8	1	0	0	0	0	4								
Cefoperazone				9	3	1	0	0	0	0	0	0	0					
Cefotaxime		12	1	0	0	0	0	0	0	0	0	0	0					
Ceftiofur					13	0	0	0	0	0	0							
Cefquinome		6	5	2	0	0	0	0	0	0	0	0	0					
Cephalexin				2	2	4	3	2	0	0	0	0	0	0	0			
Imipenem		0	0	2	5	6	0	0	0	0	0	0	0					
Tetracycline						2	0	0	0	0	0	11						
Doxycycline				0	0	0	1	2	3	6	1	0	0	0	0			
Colistin							13	0	0									
Clindamycin (Cli)			0	0	0	0	0	0	0	0	0	3	2	0	8			
Tiamulin						0	0	0	0	0	2	5	6					
Florfenicol								4	0	2	3	4						
Chloramphenicol (Chl)								2	0	0	0	7	4					
Erythromycin (Ery)		0	0	0	0	0	0	0	0	0	0	2	0	11				
Gamithromycin						0	0	0	1	1	2	9						
Tildipirosin								4	0	1	0	0	0	8				
Tylosin (Tyl)				0	0	0	0	0	0	0	0	0	0	1	2	10		
Tilmicosin							0	0	0	0	1	2	10					
Tulathromycin								0	0	1	1	0	0	1	10			
Gentamicin (Gen)				0	0	0	0	2	5	4	1	1						
Neomycin (Neo)					0	0	0	0	0	2	3	1	1	5	1			
Streptomycin (Str)						0	0	0	0	0	0	0	1	0	3	4	3	2
Enrofloxacin		6	0	0	5	2	0	0										
Marbofloxacin	0	0	6	0	0	5	1	1	0	0	0	0						
	**0.008**	**0.015**	**0.03**	**0.06**	**0.12**	**0.25**	**0.5**	**1**	**2**	**4**	**8**	**16**	**32**	**64**	**128**	**256**	**512**	**1,024**
Ciprofloxacin	4	2	0	2	4	1	0	0	0	0	0	0						
Nalidixic acid (Nal)				0	0	0	0	2	4	0	0	0	0	0	5	2		
Sulfisoxazole (Sul)				0	0	0	0	0	0	0	0	0	0	0	0	13		
Trimethoprim/ sulfamethoxazole (1:19)					11	0	1	0	0	1								

^a^The black areas indicate the concentrations not included in the test panels. Isolates that showed no growth in any of the concentrations were given the lowest MIC value. Isolates with growth in all tested concentrations were given the next serially higher MIC value above the highest tested concentration. Isolates were classified as susceptible (light gray), intermediate (middle gray), or resistant (dark gray), if breakpoints were available in the CLSI document VET01S-Ed6 (CLSI, 2023). Despite the lack of breakpoints, isolates that showed the following high MIC values were considered resistant: Ery (≥64 mg/L), Tyl (≥256 mg/L), Cli (≥128 mg/L), Str (≥32 mg/L), Neo (≥16 mg/L), Gen (≥4 mg/L), Chl (≥16 mg/L), Sul (≥256 mg/L), and Nal (≥128 mg/L).

^b^The MIC values of amoxicillin-clavulanic acid (2:1) and trimethoprim-sulfamethoxazole (1:19) are expressed as the MIC values of amoxicillin and trimethoprim, respectively.

### Detection of AMR-mediating genes, point mutations, and ICEs in *M. haemolytica*

**Figure 2:**
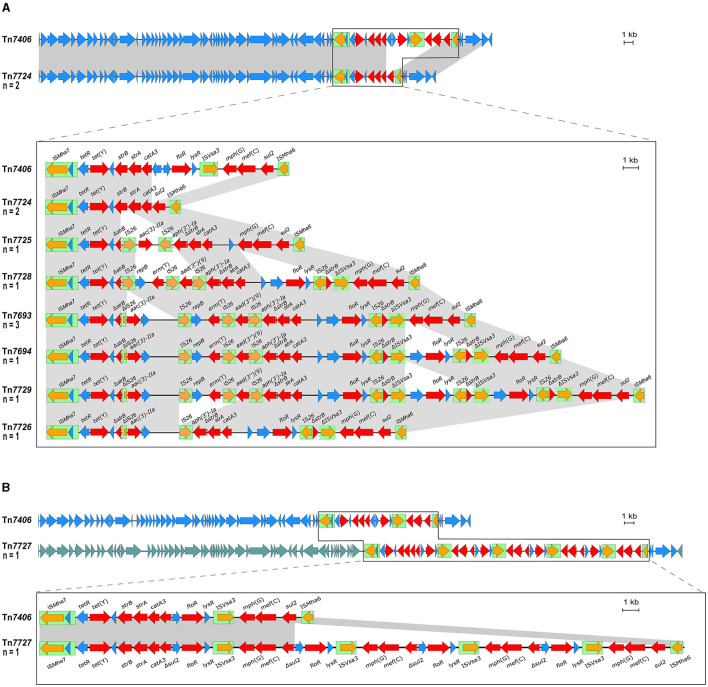
Organization of the resistance gene regions of ICEs found among the *M. haemolytica* isolates included in this study (GenBank Bioproject accession no. PRJNA960937) aligned to the sequence of the ICE Tn*7406* (GenBank accession no. CP087379). Open reading frames are shown as arrows with the arrowhead indicating the direction of transcription; resistance genes are marked in red, transposase genes in orange; other genes and genes with unknown functions in blue; IS elements are shown as green boxes; homologous regions between sequences are indicated by gray shading. Size scales are given at the right-hand side; **(A)** ICEs harboring a homologous backbone as Tn*7406*, but differing resistance regions; **(B)** Tn*7727* harboring a different set of backbone genes as Tn*7406* indicated by different blue shadings of the reading frames and comparison of the ICEs' resistance regions.

As mentioned above, two *M. haemolytica* isolates carrying novel MDR-mediating ICEs, designated Tn*7406* and Tn*7693*, have been described in Germany since 2022 (Schink et al., 2022; Kostova et al., 2023). Both ICEs had an almost identical structure with regard to their core genes with an identity of 99.8%, while their respective resistance regions only shared 55.7% identity. In this study, seven novel ICEs were identified among 11 of the 13 *M*. *haemolytica* isolates investigated. All but one of them were closely related to the two previously described ICEs (Schink et al., 2022; Kostova et al., 2023) considering their core genes, but they differed to varying degrees in their resistance regions (Figure 2A). The novel ICEs ranged in size between 51,237 and 85,505 bp. They were flanked by 11-bp direct repeats (5′-GATTCAAAATC-3′) and had inserted into a chromosomal tRNA^Leu^ copy. The ICE designated Tn*7693*, already described in Kostova et al. (2023), was found in three isolates (isolates nos. 5, 9, and 10), while the ICE Tn*7724* was identified in two isolates (isolates nos. 2 and 13). Sequence analysis revealed that the ICE Tn*7724* could be detected mainly in its circular intermediate form in isolate 13. The remaining ICEs were present only in individual isolates (Table 3). Furthermore, we were able to detect 13 different complete AMR genes among these 11 ICE-carrying *M*. *haemolytica* isolates (Table 2). Twelve of these genes were located in varying combinations within a single resistance region as part of the different ICEs described above. Overall, the 11 isolates carried five to 11 different AMR genes within their ICEs' resistance regions. With further regard to macrolide resistance, the resistance genes *mef* (C)-*mph*(G) and *erm*(T) were found in six of these isolates, while three other isolates carried only the gene combination *mef* (C)-*mph*(G). Moreover, the genes *tet*(Y), *strB, strA, catA3, sul2* occurred mostly in combination, however, in several cases only segments of the resistance gene *strB* were found. The genes *floR, aad(3“)(9), aac(3)-IIa*, and *aph(3‘)-Ia* were also identified within the ICEs. Notably, two ICEs carried more than one copy of the same *floR* gene. All but one of the detected ICEs harbored a backbone of core genes with 100% identity. The ICE designated Tn*7727* showed only 60% identity to the core gene region of the other ICEs (Figure 2B). However, the resistance region of this particular isolate was closely related to the resistance region of Tn*7406*, except that it carried three additional copies of complete *floR, mph*(G), and *mef* (C) genes and a partial *sul2* gene, respectively (Figure 2B). All copies of *floR* on Tn*7727* differed from the *floR* genes on the other ICEs in mutational changes resulting in the amino acid substitutions M32I and I43N. In addition, four of the isolates carried a small plasmid of 4,613 bp (GenBank accession number CP123959.1) containing a single *bla*_ROB − 1_ gene for β-lactam resistance, as previously described in Kostova et al. (2023). However, two isolates did not harbor any AMR genes, yet a macrolide resistance-mediating point mutation A2058G was found affecting all six copies of the 23S rRNA (Vester and Douthwaite, 2001). Moreover, a streptomycin resistance-mediating point mutation in the *rpsL* gene was detected in two isolates that resulted in the amino acid substitution K43R (Sreevatsan et al., 1996; Barnard et al., 2010; Sun et al., 2010; Dai et al., 2021). A nalidixic acid resistance-conferring point mutation in the *gyrA* gene resulted in the amino acid substitution D87Y (Ozawa et al., 2009; Table 3).

**Table 2:** AMR genes harbored by the *M. haemolytica* isolates included in the study.

**Resistance to:**	**Gene**	**No. of isolates**
β-Lactams	*bla*_ROB − 1_	4
Tetracyclines	*tet*(Y)	11
Macrolides	*mef*(C)	9
	*mph*(G)	9
Macrolides, lincosamides, streptogramin B	*erm*(T)	6
Aminoglycosides	*strA* (streptomycin)	11
	*strB* (streptomycin)	3
	*aad(3“)(9)* (presumably streptomycin)	6
	*aph(3‘)-Ia* (kanamycin, neomycin)	8
	*aac(3)-IIa* (gentamicin)	7
Phenicols	*floR* (florfenicol, chloramphenicol)	8
	*catA3* (non-fluorinated phenicols)	11
Sulfonamides	*sul2*	11

**Table 3:** Resistance profiles of the 13 *M*. *haemolytica* isolates investigated in this study.

**ID**	**Year**	**Serotype**	**MLST**	**ICE (size)**	**Resistance phenotype^a^**	**Resistance genotype**
1	2009	1	ST1	-	Ery, Tilm, Tdp, Gam, Tul, Tyl, Cli, Sul, Str, Tia	no AMR genes detected^b, d^
2	2012	2	ST4	Tn*7724* (51,237 bp)	Tilm, Tyl, Str, Chl, Sul, Tet	*strA, strB, catA3, sul2, tet*(Y)
3	2013	2	ST4	Tn*7725* (59,217 bp)	Ery, Gam, Tul, Tyl, Str, Gen, Neo, Chl, Sul, Tet	*mef*(C), *mph*(G)*, strA, aac(3)-IIa, aph(3‘)-Ia, catA3, sul2, tet*(Y)
4	2017	1	ST1	Tn*7726* (65,125 bp)	Ery, Tilm, Gam, Tul, Tyl, Str, Gen, Neo, Chl, Sul, Tet, Nal	*mef*(C)*, mph*(G)*, strA, aac(3)-IIa, aph(3‘)-Ia, catA3, floR, sul2, tet*(Y)^c^
5	2017	1	ST1	Tn*7693* (69,571 bp)	Ery, Tilm, Tdp, Gam, Tul, Tyl, Cli, Str, Neo, Chl, Ffn, Sul, Tet, Nal	*erm*(T)*, mef*(C)*, mph*(G)*, strA, aac(3)-IIa, aph(3‘)-Ia, catA3, floR, sul2, tet*(Y)^c^
6	2018	2	ST4	Tn*7727* (85,505 bp)	Ery, Gam, Tul, Tyl, Str, Chl, Ffn, Sul, Tet, Tia	*mef*(C)*, mph*(G)*, strA, strB, catA3, floR, sul2, tet*(Y)
7	2018	1	ST1	-	Ery, Tilm, Tdp, Gam, Tul, Tyl, Cli, Sul, Str, Tia	no AMR genes detected^b, d^
8	2019	1	ST1	Tn*7728* (65,981 bp)	Ery, Tilm, Tdp, Gam, Tul, Tyl, Cli, Str, Neo, Chl, Ffn, Sul, Tet, Nal, Tia	*erm*(T)*, mef*(C)*, mph*(G)*, strA, aph(3‘)-Ia, catA3, floR, sul2, tet*(Y)^c^
9	2019	1	ST1	Tn*7693* (69,571 bp)	Ery, Tilm, Tdp, Gam, Tul, Tyl, Cli, Str, Gen, Neo, Chl, Ffn, Sul, Tet, Pen, Amp, Nal	*erm*(T)*, mef*(C)*, mph*(G)*, strA, aac(3)-IIa, aph(3‘)-Ia, catA3, floR, sul2, tet*(Y), $bla_{\text{ROB }-\;{\text{1}}^{\text{c}}}$
10	2019	1	ST1	Tn*7693* (69,571 bp)	Ery, Tilm, Tdp, Gam, Tul, Tyl, Cli, Str, Gen, Neo, Chl, Ffn, Sul, Tet, Pen, Amp, Nal	*erm*(T)*, mef*(C)*, mph*(G)*, strA, aac(3)-IIa, aph(3‘)-Ia, catA3, floR, sul2, tet*(Y), $bla_{\text{ROB }-\;{\text{1}}^{\text{c}}}$
11	2019	1	ST1	Tn*7729* (79,561 bp)	Ery, Tilm, Tdp, Gam, Tul, Tyl, Cli, Str, Gen, Neo, Chl, Ffn, Sul, Tet, Nal	*erm*(T)*, mef*(C)*, mph*(G)*, strA, aac(3)-IIa, aph(3‘)-Ia, catA3, floR, sul2, tet*(Y)^c^
12	2019	1	ST1	Tn*7694* (74,556 bp)	Ery, Tilm, Tdp, Gam, Tul, Tyl, Cli, Str, Gen, Neo, Chl, Ffn, Sul, Tet, Pen, Amp	*erm*(T)*, mef*(C), *mph*(G)*, strA, aac(3)-IIa, aph(3‘)-Ia, catA3, floR, sul2, tet*(Y), *bla*_ROB − 1_
13	2020	2	ST4	Tn*7724* (51,237 bp)	Tilm, Tyl, Str, Chl, Sul, Tet, Pen, Amp, Tia	*strA, strB, catA3, sul2, tet*(Y), *bla*_ROB − 1_

^a^Ery, erythromycin; Tilm, tilmicosin; Tdp, tildipirosin; Gam, gamithromycin; Tul, tulathromycin; Tyl, tylosin; Cli, clindamycin; Str, streptomycin; Neo, neomycin; Gen, gentamicin; Ffn, florfenicol; Chl, chloramphenicol; Sul, sulfisoxazole; Tet, tetracycline; Nal, nalidixic acid; Pen, penicillin; Amp, ampicillin; Tia, tiamulin. Despite the lack of clinical breakpoints approved by the CLSI, isolates that showed high MIC values of Ery (≥64 mg/L), Tyl (≥128 mg/L), Cli (≥128 mg/L), Str (≥32 mg/L), Neo (≥16 mg/L), Gen (≥4 mg/L), Chl (≥16 mg/L), Sul (≥256 mg/L), Nal (≥128 mg/L), and Tia (≥32 mg/L) were considered as resistant.

^b^Two isolates harbored macrolide resistance-mediating point mutations affecting all six copies of the 23S rRNA (A2058G).

^c^Isolates harbored a fluoroquinolone resistance-mediating point mutation in *gyrA* (D87Y).

^d^Isolates harbored a streptomycin resistance-mediating mutation in *rpsL* (K43R).

### Correlation of AMR phenotypes and genotypes of *M*. *haemolytica*

The phenotypic and genotypic AMR profiles correlated almost completely (Table 3). The macrolide resistance and/or elevated MICs in nine of the isolates correlated with the presence of the genes *mef* (C)-*mph*(G), or *mef* (C)-*mph*(G) and *erm*(T). However, it is noteworthy that the MIC values of isolates carrying the *erm*(T) gene were distinctly higher for all macrolides tested, compared with the isolates carrying only the *mef* (C)-*mph*(G) genes. Moreover, the screening for inducible macrolide/lincosamide resistance revealed a constitutive expression of the *erm*(T) gene. Furthermore, the same high MIC values were observed in the two isolates harboring a mutational change in all six copies of the 23S rRNA. Notably, two isolates did not carry any macrolide resistance-mediating genes or mutations, however, they exhibited only slightly elevated erythromycin MICs (16 mg/L), and were resistant to tilmicosin (MIC of ≥ 32 mg/L), but susceptible to tildipirosin, tulathromycin and gamithromycin. Furthermore, all isolates that harbored the *erm*(T) gene, or showed the mutational change in the 23S rRNA, respectively, also exhibited high clindamycin MIC values of ≥128 mg/L.

Regarding the β-lactam resistance, isolates were resistant to ampicillin and penicillin only when harboring the *bla*_ROB − 1_-carrying plasmid. The florfenicol MICs of the isolates carrying the *floR* gene varied when more than one copy of the gene was present on the same ICE. More precisely, isolates harboring only a single *floR* gene exhibited florfenicol MICs between 4 and 16 mg/L, whereas the isolate carrying Tn*7694* that harbored two *floR* copies exhibited a MIC of 32 mg/L and the isolate with Tn*7729* that carried three *floR* copies exhibited a MIC value of 128 mg/L. Nevertheless, isolate 6 harboring ICE Tn*7727* with even four *floR* copies showed a florfenicol MIC of only 32 mg/L. These four *floR* genes differed from the *floR* genes found in the other isolates in two mutations resulting in the amino acid substitutions M32I and I43N. Further studies are needed to analyze whether these amino acid substitutions have an impact on the florfenicol MIC. Furthermore, there was a 100% correlation between the detected high chloramphenicol MIC values and the presence of the *catA3* gene, as well as tetracycline resistance and the associated *tet*(Y) gene. Considering aminoglycosides, the elevated MICs of gentamicin correlated in almost all isolates with the presence of the *aac(3)-*IIa gene. However, isolate 5 carried the *aac(3)-*IIa gene but showed only a MIC of 2 mg/L. In contrast, one isolate, which harbored a modified *aac(3)*-IIa promotor region expressed a distinctly higher MIC value of ≥16 mg/L, suggesting that this mutational change might be involved in increased gentamicin resistance. Furthermore, the observed high streptomycin MICs correlated with the presence of the *strA* gene, and they were not distinctly higher in isolates that carried a complete *strB* gene in combination. Moreover, a streptomycin resistance-mediating point mutation in the *rpsL* gene was detected in two isolates that resulted in the amino acid substitution K43R (Sreevatsan et al., 1996; Barnard et al., 2010; Sun et al., 2010; Dai et al., 2021). In addition, the elevated MIC values of nalidixic acid correlated with a point mutation in the *gyrA* gene resulting in the amino acid substitution D87Y (Ozawa et al., 2009). Notably, the high MICs of the sulfonamide sulfisoxazole did not correlate with the presence of the *sul2* gene, as the isolates that did not carry *sul2* exhibited the same elevated MICs.

### Serotyping, MLST, and phylogenetic analysis of *M*. *haemolytica*

**Figure 3:**
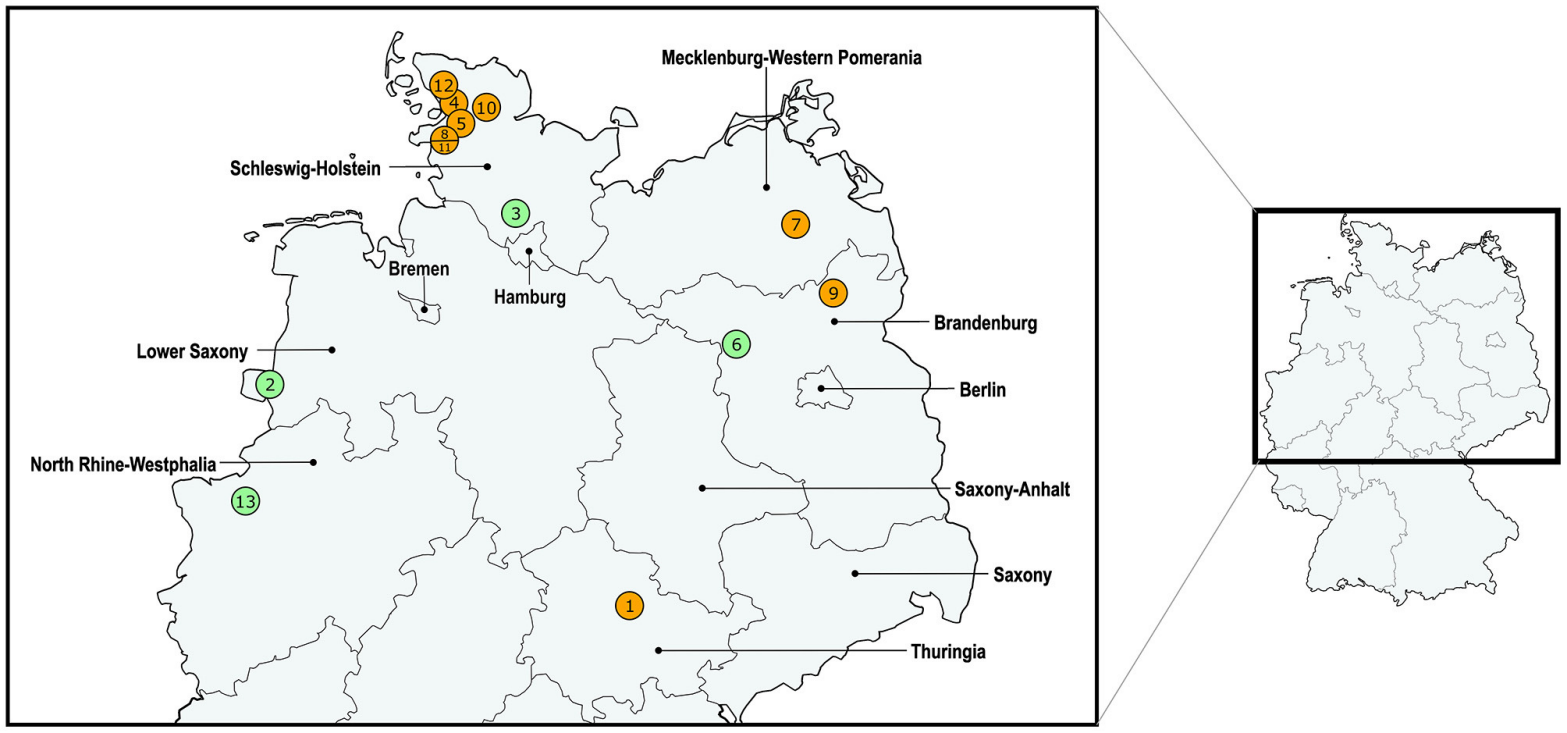
Distribution of the investigated macrolide-resistant *M*. *haemolytica* isolates included in this study. The isolate IDs are given within the circles, which contain more than one number in cases where multiple isolates were found within the same zip code area. The colors of the circles refer to the differentiation of isolate sequence types: ST1 in orange and ST4 in green.

**Figure 4:**
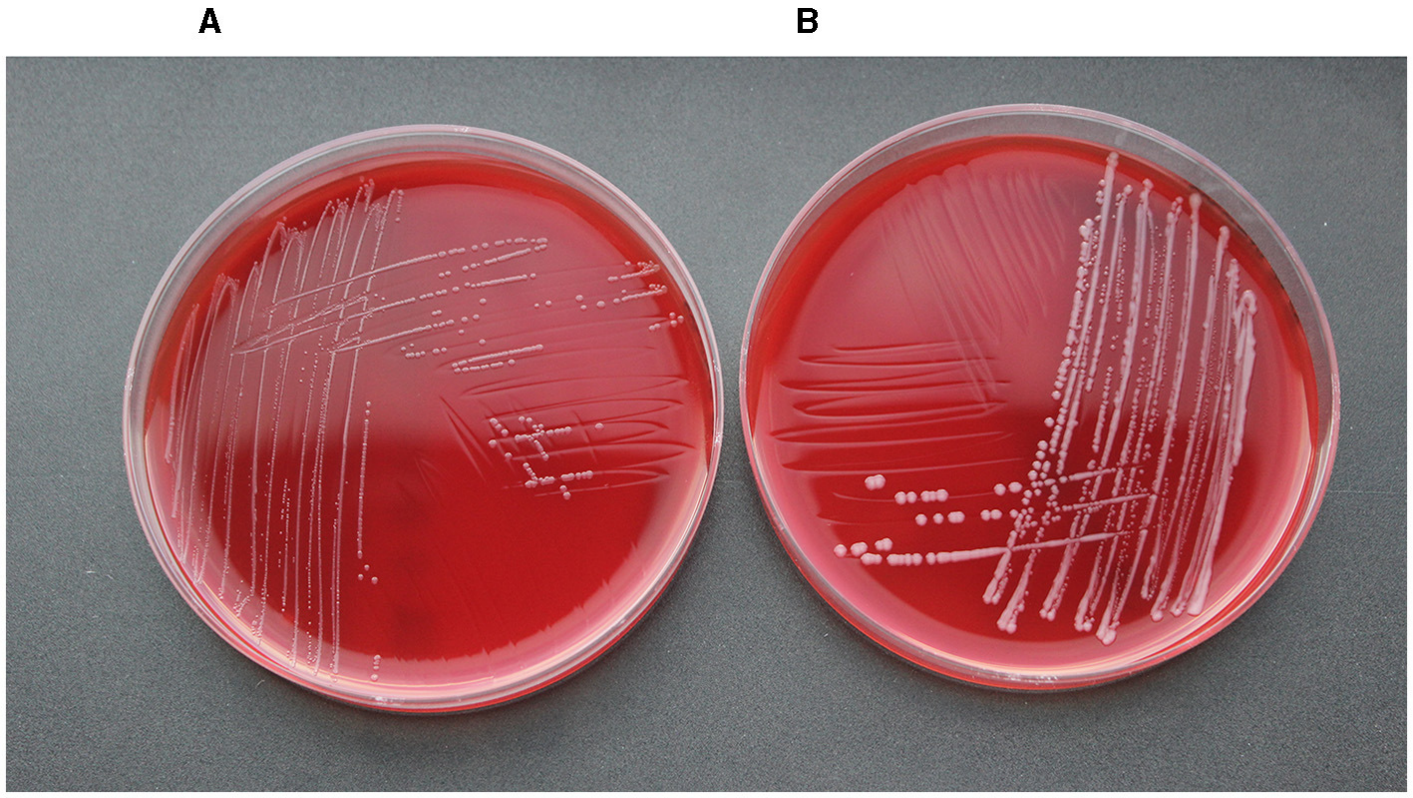
Comparison of the colony morphology of one **(A)** serotype 2 and one **(B)** serotype 1 isolate.

**Figure 5:**
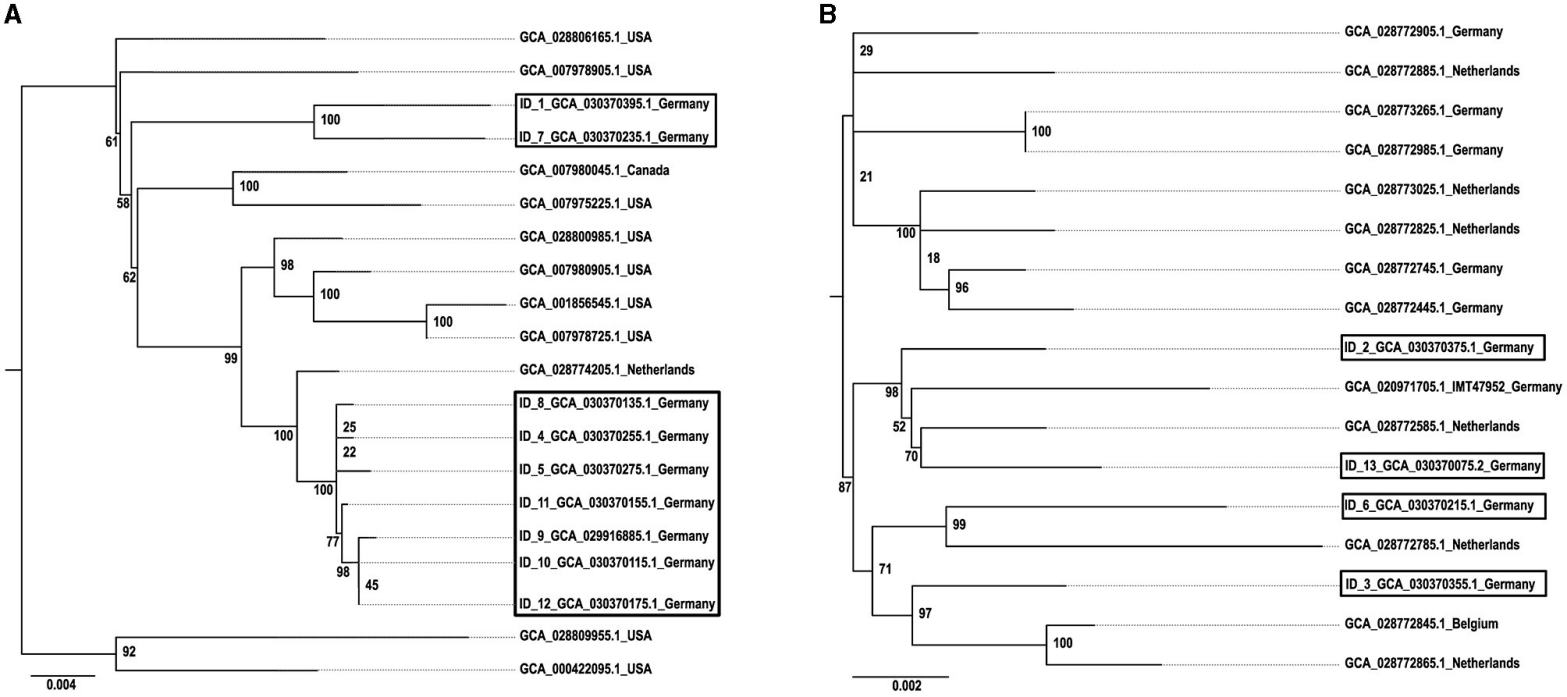
Phylogeny of **(A)** ST1 and **(B)** ST4 *M. haemolytica* isolates. The isolates obtained in this study are indicated by boxes and displayed in comparison with reference isolates. The GenBank assembly accession numbers are given for each isolate, in addition with the isolate ID for the isolates obtained within this study. The maximum likelihood trees were generated with IQ tree and visualized with FigTree v1.4.4 using the TVMe+ASC+G4 model for ST1 and the SYM+ASC+G4 model for ST4. The scale bar displays the average number of nucleotide acid substitutions per site. The numbers at the major branch points refer to the percentage of times that a particular node was found in 10,000 bootstrap replications.

Figure 3 shows the geographical distribution of the investigated macrolide-resistant *M. haemolytica* isolates within Germany. Most of them (*n* = 7) originated from the federal state Schleswig-Holstein, followed by Brandenburg (*n* = 2), and North Rhine-Westphalia, Mecklenburg-Western Pomerania, Lower Saxony and Thuringia (*n* = 1, each). Furthermore, the most prevalent *M*. *haemolytica* serotype in our study was serotype 1 (*n* = 9), while the remaining four isolates belonged to serotype 2. Notably, we observed a phenotypic difference in the morphology of the colonies between the two serotypes. While colonies of serotype 1 appeared often bigger and mucoid, the colonies of serotype 2 were smaller and less mucoid (Figure 4). The MLST analysis of the 13 genomes revealed two major sequence types, ST1 and ST4, for which separate core genomes were generated (Figure 5). The ST1 isolates had a core genome length of 1,900,882 bp with a total of 469 SNPs (Supplementary material 2). Out of 3,163 genes, a total of 2,225 genes were identified as core genes (present in ≥99% of the isolates), 140 as soft core genes (present in ≥95% and < 99% of the isolates), 558 as shell genes (present in ≥15% and < 95% of the isolates), and 240 as cloud genes (present in < 15% of the isolates). The ST4 isolates had a core genome length of 1,856,620 bp with a total of 316 SNPs (Supplementary material 2). For the ST4 isolates, out of 2,814 gene sequences in total, 2,142 sequences were identified as core genes, 0 as soft core genes, 546 as shell genes, and 126 as cloud genes. As described previously, ST1 and ST4 are distantly related (het Lam et al., 2023), with distinctly different allelic profiles (ST1 *adk*_1, *aroE*_2, *deoD*_1, *gapDH*_2, *gnd*_1, *mdh*_2, *zwf* _1; ST4 *adk*_2, *aroE*_1, *deoD*_1, *gapDH*_3, *gnd*_2, *mdh*_1, *zwf* _2). The phylogenetic analysis revealed a very close relationship between seven of the ST1 isolates with only up to 13 SNPs (Supplementary material 2). Interestingly, six of these isolates (isolates nos. 4, 5, 8, 10, 11, and 12) were collected in close proximity to each other within the federal state Schleswig-Holstein, with two isolates even originating from the same zip code region (Figure 3). The remaining isolate was collected in Brandenburg. The other two ST1 isolates, nos. 1 and 7, were from Thuringia and Mecklenburg-Western Pomerania, respectively, and showed 64 SNPs difference to one another. Yet, there was higher genomic difference to the aforementioned isolates from Schleswig-Holstein and Brandenburg with SNPs varying from 99 to 108. The ST4 isolates did not show a very close relationship (31–71 SNPs).

## Discussion

Previous studies on the genetics of macrolide resistance in bovine *M. haemolytica* isolates from Germany focused on single isolates (Schink et al., 2022; Kostova et al., 2023). In this study, we investigated a larger number of macrolide-resistant BRD-associated *M. haemolytica* isolates detected since 2009 in the German national resistance monitoring program GE*RM*-Vet. It is important to note here that GE*RM*-Vet only includes isolates from diseased animals which are provided by diagnostic laboratories on a voluntary basis according to annually varying sampling plans. Therefore, the program might not mirror the true prevalence of the monitored pathogens, but it gives an overview of the current AMR situation in bacterial pathogens from diseased animals in Germany including trends over time regarding resistances to specific antimicrobial agents. A pan-European survey conducted in 2016 reported that the susceptibility to antimicrobial agents in BRD-associated organisms is high for almost all agents licensed for the treatment in the European Union. In particular, resistances to gamithromycin and tulathromycin were reported in < 3% of the isolates investigated (el Garch et al., 2016). In contrast to the situation in Europe, studies from North America show that an increase in AMR has been reported for BRD-associated *M. haemolytica* for the last two decades (Klima et al., 2020; Andrés-Lasheras et al., 2022). Although the GE*RM*-Vet data suggests that the situation in Germany is still very favorable considering AMR in *M*. *haemolytica* from cattle, the possibly rising proportion of macrolide-resistant isolates in Germany needs further monitoring. Macrolides belong to the “critically important antimicrobials” for human medicine as defined by the World Health Organization (WHO).^14^ The Antimicrobial Advice *ad hoc* Expert Group (AMEG) of the European Medicines Agency (EMA) has categorized antimicrobial agents for the application to animals with regard to prudent and responsible use. Here, macrolides are part of category C and should, therefore, be prescribed and/or used with caution.^15^ Nevertheless, macrolides are also often used for metaphylactic treatment of cattle with BRD (Younes et al., 2022). In contrast to prophylaxis where medical products are administered to an animal or group of animals without an established diagnosis of a clinical disease, the metaphylactic treatment is carried out to a whole group of animals at a point in time when only a minority of the animals suffer from a disease. The aim of metaphylaxis is not only to treat the clinically sick animals but also those at risk or in contact with the sick animals to control the spread of the causative pathogen (Baptiste and Pokludová, 2020). However, it has been suggested that the metaphylactic treatment of cattle with antimicrobial agents plays a considerable role in decreasing the susceptibility of *M. haemolytica* (Younes et al., 2022). Moreover, the metaphylactic treatment with any antimicrobial compound may select for acquisition of AMR and/or MGEs, such as ICEs. For this reason, applying the principles of antimicrobial stewardship is of utmost importance for safeguarding the effectiveness of antimicrobial drugs. In addition, maintenance of healthy and less susceptible animals is essential in the management of the multifactorial BRD by implementing measures, such as improved animal husbandry, adequate colostrum intake and nutrition, reduction of stress, e.g., from transportation, identification and removal of persistently infected BRD cattle and prevention of infections trough vaccines.^16^ Specifically in Germany, different vaccines are available containing bovine respiratory syncytial virus, parainfluenza-3 virus, bovine viral diarrhea virus, *Histophilus somni* and/or *M. haemolytica* as vaccine antigen(s) in differing combinations. The German veterinary vaccination commission (StIKo Vet) recommends an early immunization with one of the combination vaccines of calves born and raised in stables for fattening, but also on dairy farms where problems with BRD have occurred. For farms where in particular *Mannheimia* spp. have been shown to be a problem, additional combination or single vaccines containing *M. haemolytica* components are available.^17^ Nevertheless, antimicrobial treatments against BRD will be necessary even in the best-managed animal production systems. In this case, pathogen identification and subsequent AST, early treatment including consideration of non-steroidal anti-inflammatory drugs, isolation and monitoring of diseased or high-risk animals, and close collaboration between farmer and veterinarian should have priority (see text footnote 16).

Elevated MIC values of the different antimicrobial agents tested in this study were highly correlated with either the presence of AMR genes or AMR-mediating mutations in the investigated 13 bovine *M*. *haemolytica* isolates. High sulfisoxazole MICs did not correlate with the presence of the *sul2* gene, since also isolates not carrying this gene showed these high values. A possible reason for this observation needs to be investigated further. However, Gram-negative bacteria are intrinsically resistant to several classes of antimicrobial agents, mostly due to an impermeability of their outer membrane and the presence and activity of efflux pumps (Melander et al., 2023). For this reason, *M*. *haemolytica* as a Gram-negative pathogen may also be intrinsically resistant to sulfisoxazole. Since sulfonamides are usually only used as potentiated preparations in combination with trimethoprim, ormetoprim or pyrimethamine,^18^ and *M. haemolytica* isolates are commonly susceptible to these diaminopyrimidines, this potential intrinsic sulfonamide resistance of *M*. *haemolytica* seems to be of minor relevance for clinical practice.

Furthermore, 10 of the 13 isolates showed varying MDR profiles. A trend toward increasing MDR has also been observed in another recent study that revealed MDR in 5.13% of *M. haemolytica* and 13.91% of *Pasteurella multocida* isolates from cattle with BRD in Bavaria, Germany investigated over the years 2015 to 2020 (Melchner et al., 2021). Notably, the interpretation of the MIC values and classification of the isolates was difficult due to the lack of CLSI-approved clinical breakpoints for 22 of the 31 antimicrobial agents tested. This underlines the urgent need to establish clinical breakpoints for further antimicrobial agents, as previously stated in el Garch et al. (2016). Considering the genetic basis of AMR, 11 of the 13 *M*. *haemolytica* isolates harbored eight similar ICEs carrying up to 11 different AMR genes. MDR-mediating ICEs are widely spread and already known in BRD-associated *Pasteurellacae* from North America, with *erm*(42), *mrs*(E), and *mph*(E) as commonly detected macrolide resistance genes (Brenner Michael et al., 2011a,b; Klima et al., 2014b). In contrast, this study showed that the most prevalent macrolide resistance genes in Germany were *mef* (C)-*mph*(G) and *erm*(T). We observed an increase in the MIC values of (i) gamithromycin in the presence of the resistance genes *mef* (C)-*mph*(G) and (ii) tildipirosin in the presence of *erm*(T). A similar observation was made by Brenner Michael et al. (2012) who noted increases in the gamithromycin or tildipirosin MICs when the resistance genes *mrs*(E)-*mph*(E) or *erm*(42), respectively, were present. Considering the ability of ICEs that carry a large number of different AMR genes to transfer themselves to other host bacteria, there may be a distinct increase in MDR, including resistance to macrolides in *Pasteurellaceae* from cattle, in Germany in the future, following examples from abroad (Credille, 2020). The location of multiple AMR genes on a single MGE, such as an ICE, may increase the risk of co-selection processes. Therefore, resistance to several antimicrobial classes may be transferred in a single transfer event, even under the selection pressure of a single antimicrobial agent. The ICEs described among the *M*. *haemolytica* isolates in this study had in all but one case an almost identical core structure, but differed in their resistance regions. This is not surprising since it is already known that the diversity of ICEs is often based on differences within their resistance regions (Klima et al., 2020). The presence of identical ICE types in more than one isolate as well as distinct structural similarities of their resistance regions despite the overall differences (Figure 2) already point toward horizontal gene transfer processes of MDR-mediating ICEs among bovine *M*. *haemolytica* in Germany. The structural similarities of the different ICE types described here refer to repetitions of certain segments and/or the presence of truncated genes. This suggests genetic rearrangements that resulted in the emergence of novel ICE variants that are similar to already existing types. This might indicate an ongoing evolution of ICEs among bovine *M. haemolytica*, which is, taking into account horizontal gene transfer mechanisms, surely not restricted to this bacterial genus. Since it has already been reported that a transfer of MDR-mediating ICEs between different BRD pathogens is likely (Brenner Michael et al., 2011a,b), follow-up research on AMR in, for example, *P*. *multocida* isolates from cattle collected in Germany is needed to evaluate if there is evidence for cross-genus transfer of MDR-mediating ICEs among different BRD pathogens in German cattle *in-vivo*. Under *in-vitro* conditions, such a transfer has already been confirmed (Brenner Michael et al., 2011b). In addition, an AMR gene-carrying plasmid was also detected within our isolate collection, which adds to the risk of AMR dissemination via horizontal gene transfer of MGEs other than ICEs among bovine *M*. *haemolytica*.

The most prevalent *M*. *haemolytica* serotype that we identified in this study was serotype 1, but also serotype 2 was detected. In Europe, the dominant *M*. *haemolytica* serotypes are 1, 2, and 6. Serotypes 1 and 6 are often associated with BRD, while serotype 2 is considered as less virulent and also present in the nasopharynx of healthy bovines (Klima et al., 2014a; Andrés-Lasheras et al., 2019). The phenotypic difference in the morphology of the colonies that we observed between the two serotypes (Figure 4) might be related to the capsular structure of the two different serotypes. A previous study revealed that the capsular polysaccharide of *M*. *haemolytica* serotype 1 is composed of N-acetylmannosaminuronic acid and N-acetylmannosamine repeats, while the capsule of serotype 2 consists of a linear polymer of N-acetylneuraminic acid (Neu5Ac) with α(2–8) linkages (Barrallo et al., 1999; Lo et al., 2001).

Moreover, the phylogenetic analysis suggests a higher genomic correlation between isolates originating from nearby regions. However, due to the small number of isolates included in this study, this needs further monitoring. Considering that all ST1 reference genomes to date originate from the USA, it is necessary to expand the *M. haemolytica* database by collecting more WGS data to get a more comprehensive picture of circulating clones worldwide. In addition, in this study ST1 correlated 100% with serotype 1 and ST4 with serotype 2 (Table 3), even though other studies demonstrated that there is no correlation between ST and serotype (Christensen et al., 2021). A reason for this observation might also be the comparatively small number of isolates included in this study.

Finally, the bovine *M*. *haemolytica* isolates investigated in this study already displayed AMR to multiple classes of therapeutically relevant antimicrobial agents, such as phenicols, penicillins, tetracyclines, and macrolides, although MDR in *M. haemolytica* isolates from Germany is still a rare phenomenon compared to other regions, such as North America. This highlights the increasing risk of limited therapeutic options for the BRD management in the future. In addition, the isolates investigated in this study originated only from diseased cattle and there is no resistance data available regarding *M*. *haemolytica* isolates from healthy colonized animals. Therefore, the risk imposed by AMR in bovine *M*. *haemolytica* from Germany might be underestimated. In order to address future prognosis on the AMR prevalence in BRD-associated pathogens in cattle herds, joint efforts by farmers, veterinarians and scientists are required. Supporting information, such as the size of the feedlot, the origin of the animals (auction vs. direct sourced), the shipment conditions, the general health conditions of the animals as well as previous diseases and treatments with antimicrobial agents are important to manage a multifactorial disease, such as BRD. Nevertheless, pathogen identification and subsequent AST still remain the most essential tools to ensure the efficacy of the antimicrobial agents applied to control BRD in cattle herds.

## Data availability statement

The datasets presented in this study can be found in online repositories. The names of the repository/repositories and accession number(s) can be found in the article/Supplementary material.

## Author contributions

VK: Data curation, Formal analysis, Investigation, Methodology, Validation, Visualization, Writing – original draft, Writing – review & editing. DH: Data curation, Formal analysis, Investigation, Methodology, Software, Validation, Visualization, Writing – review & editing. HK: Conceptualization, Data curation, Resources, Writing – review & editing. SF: Data curation, Resources, Writing – review & editing. SS: Conceptualization, Formal analysis, Funding acquisition, Investigation, Methodology, Project administration, Resources, Supervision, Validation, Writing – original draft, Writing – review & editing. HK-H: Conceptualization, Formal analysis, Investigation, Methodology, Project administration, Supervision, Validation, Writing – original draft, Writing – review & editing.

## Footnotes

^1^ https://www.bmel-statistik.de/landwirtschaft/tierhaltung/rinderhaltung

^2^ https://www.accc.gov.au/system/files/1128%20Beef%20and%20cattle%20report_D08.pdf

^3^ https://www.bvl.bund.de/SharedDocs/Berichte/07_Resistenzmonitoringstudie/Bericht_Resistenzmonitoring_2021.pdf?__blob=publicationFile&v=2

^4^ https://www.bioinformatics.babraham.ac.uk/projects/fastqc/

^5^ https://github.com/rrwick/Porechop

^6^ https://github.com/rrwick/Filtlong

^7^ https://github.com/yfukasawa/LongQC

^8^ https://transposon.lstmed.ac.uk/

^9^https://ivsmlst.sund.ku.dk/, https://pubmlst.org/organisms/mannheimia-haemolytica

^10^ https://www.ncbi.nlm.nih.gov/

^11^ http://tree.bio.ed.ac.uk/software/figtree/

^12^ https://github.com/tseemann/abricate

^13^ http://www-is.biotoul.fr

^14^ https://cdn.who.int/media/docs/default-source/antimicrobial-resistance/amr-gcp-irc/who_mialist_draft_forexternaldiscussion.pdf?sfvrsn=af6f2ebf_1

^15^ https://www.ema.europa.eu/en/documents/report/infographic-categorisation-antibiotics-use-animals-prudent-and-responsible-use_en.pdf

^16^ https://www.cdfa.ca.gov/AHFSS/AUS/docs/AUS-CowCalf-BRD-Infographic.pdf

^17^ https://www.openagrar.de/servlets/MCRFileNodeServlet/openagrar_derivate_00044277/Impfleitlinie_Wiederkaeuer_2022-01-01_ba.pdf

^18^ https://www.msdvetmanual.com/pharmacology/antibacterial-agents/sulfonamides-and-sulfonamide-combinations-use-in-animals
